# Poly-γ-glutamic Acid Synthesis, Gene Regulation, Phylogenetic Relationships, and Role in Fermentation

**DOI:** 10.3390/ijms18122644

**Published:** 2017-12-07

**Authors:** Yi-Huang Hsueh, Kai-Yao Huang, Sikhumbuzo Charles Kunene, Tzong-Yi Lee

**Affiliations:** 1 Graduate School of Biotechnology and Bioengineering, Yuan Ze University, Taoyuan city 32003, Taiwan; s1055811@mail.yzu.edu.tw; 2 Department of Computer Science and Engineering, Yuan Ze University, Taoyuan city 32003, Taiwan; kaiyao.tw@gmail.com (K.-Y.H.); francis@saturn.yzu.edu.tw (T.-Y.L.); 3 Department of Medical Research, Hsinchu Mackay Memorial Hospital, Hsinchu city 300, Taiwan

**Keywords:** poly-γ-glutamic acid, *Bacillus* species, phylogenetic analysis, fermentation

## Abstract

Poly-γ-glutamic acid (γ-PGA) is a biodegradable biopolymer produced by several bacteria, including *Bacillus subtilis* and other *Bacillus* species; it has good biocompatibility, is non-toxic, and has various potential biological applications in the food, pharmaceutical, cosmetic, and other industries. In this review, we have described the mechanisms of γ-PGA synthesis and gene regulation, its role in fermentation, and the phylogenetic relationships among various *pgsBCAE*, a biosynthesis gene cluster of γ-PGA, and *pgdS*, a degradation gene of γ-PGA. We also discuss potential applications of γ-PGA and highlight the established genetic recombinant bacterial strains that produce high levels of γ-PGA, which can be useful for large-scale γ-PGA production.

## 1. Introduction

Poly-γ-glutamic acid (γ-PGA) is a natural anionic biopolymer without a fixed molecular weight comprised of only glutamic acid residues [1]. It is water soluble and biodegradable and has good thickening capacity, excellent absorbability, and high metal-binding capacity [2]. Moreover, γ-PGA has multiple potential applications—as a drug or gene carrier in medicine [3,4,5], as a food stabilizer, nutritional aid, and food additive in the food industry [6,7,8], for the development of plastics, and for recovery of heavy metal ions [9,10].

γ-PGA is produced by bacteria, especially *Bacillus* species. It was first identified in 1937 from the capsule of *Bacillus anthracis*, which is one of the causative agents of anthrax [11]. Other naturally occurring sources of γ-PGA include the mucilage of *natto* and *chungkookjang*, which are traditional Japanese and Korean foods, respectively, made from soybeans fermented with *Bacillus subtilis* subsp. *natto* and *chungkookjang* [12,13]. γ-PGA is polymerized as a d- and l-glutamate polymer via formation of a peptide bond between the α- amino and γ-carboxyl groups. The molecular weight is typically between 10 and 1000 kDa, although it can sometimes exceed 2000 kDa [12,14]. In this review, we have described mechanisms underlying γ-PGA synthesis, regulatory genes of γ-PGA and their phylogenetic relationships, and potential applications of γ-PGA.

## 2. γ-PGA Producers

γ-PGA is the natural form of PGA that is biosynthesized at the onset of the stationary growth phase due to nutrient starvation/limitation during this phase [15,16]. However, γ-PGA can also be produced in laboratories or by industrial fermentation. γ-PGA synthesis requires glutamate, which is produced via two pathways. The most common is through the tricarboxylic acid (TCA) cycle, whereby glucose and pyruvate is transformed into α-ketoglutarate, which is then converted to l-glutamic acid. A second pathway utilizes extracellular l-glutamic acid synthesized from α-ketoglutaric acid and ammonium sulfate, which is catalyzed by glutamate dehydrogenase in the absence of glutamine. In the presence of l-glutamine, the synthesis is catalyzed by glutamine synthetase and glutamine-2-oxoglutarate aminotransferase [17]. This might increase the amount of glutamic acid in the cell and then cause an increase in PGA production.

**Figure 1:**
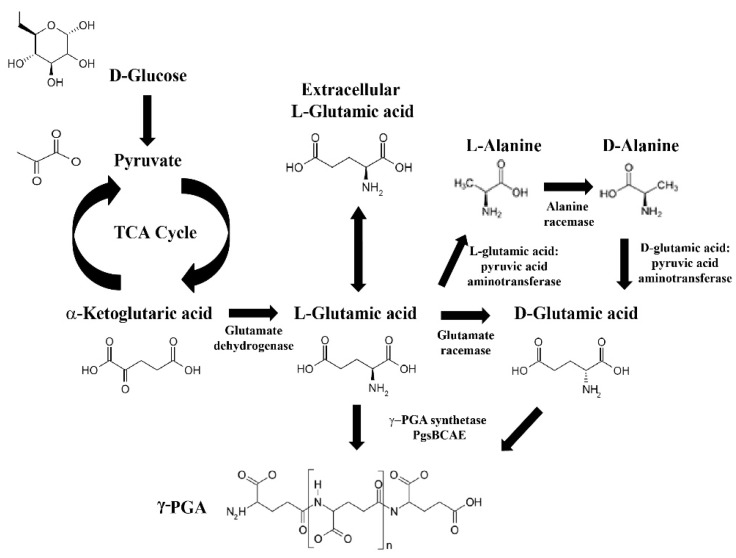
Routes of poly-γ-glutamic acid (γ-PGA) synthesis. Arrows: demonstrate the proceeding directions of the reaction. Bi-directional arrows reflect the chemical reaction is reversible. Arced arrows: represent the reaction cycle.

The mechanism underlying racemization of l-glutamic acid to d-glutamic acid has been presented by several researchers [18,19]. d-glutamic acid is formed from l-glutamic acid whereby l-glutamic acid, pyruvic acid aminotransferase catalyzes the conversion of l-glutamic acid to l-alanine. l-alanine is then racemized into d-alanine via alanine racemase. d-alanine is then converted to d-glutamic acid using d-glutamic, pyruvic acid aminotransferase [20]. l-glutamic acid and d-glutamic acid are then incorporated into the growing γ-PGA polymer. The routes of γ-PGA synthesis are summarized in Figure 1. 

The mechanism of polymerization is shown to be adenosine triphosphate (ATP) dependent; first, a terminal carboxyl group of the γ-PGA chain accepts the phosphoryl group transferred from the gamma phosphate of ATP. Second, the amino group of glutamic acid acts as a donor for nucleophilic and interacts with the phosphorylated carboxyl group, resulting in the formation of an amide linkage and elongation of the γ-PGA chain by a glutamic acid residue [21].

γ-PGA is mainly produced by gram-positive bacteria belonging to the genus *Bacillus*, including *B. subtilis*, *B. subtilis* subsp. *natto*, and *B. licheniformis* [13,19,22] and is secreted into the extracellular milieu, where it serves as a source of energy for microorganisms [23]. Other gram-positive species, such as *Staphylococcus epidermidis* and *B. anthracis*, synthesize γ-PGA that remains bound to the cell wall as a capsule component that enables immune evasion [15,23]. In addition, some Archaea, such as *Natrialba asiatica*, produce γ-PGA, which reduces the salt concentration of the surrounding environment [24].

The molecular weight and d-/l-glutamic acid ratio of γ-PGA are dependent on the species of microorganism and growth conditions [25]. For example, *B. anthracis* mainly produces d-glutamic acid-type γ-PGA (γ-(d)-PGA); *N. aegyptiaca* produces an extremely long form of γ-(l)-PGA [11,24]; *S. epidermidis* produces γ-(d,l)-PGA at a ratio of 40% (d) and 60% (l) [23]; *B. licheniformis* produces various types of γ-(d,l)-PGA, with d-glutamate contents ranging from 10% to 100% [25,26]; and *B. megaterium* produces γ-PGA with d and l contents of 30% and 70% [27], respectively (Table 1).

## 3. Genes Involved in γ-PGA Synthesis and Degradation

**Figure 2:**
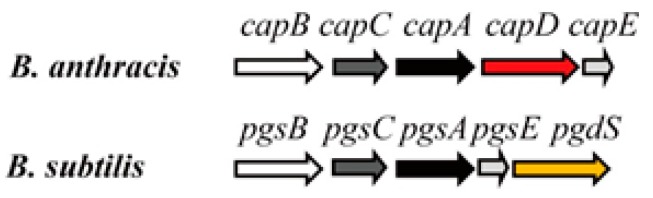
*pgsBCA* and *capBCA* gene clusters.

The γ-PGA synthesis genes *pgsB*, *pgsC*, *pgsA*, and *pgsE* were first identified in *B. subtilis* and *B. anthracis* [28,30,31]. Deletion of *pgsB*, *pgsC*, or *pgsA*, which are located in the same operon (Figure 2), blocks γ-PGA synthesis in bacterial cells. *pgsE*, which is downstream of *pgsA* (also known as *ywtC*), has low homology with *B. anthracis capE* and may also be involved in γ-PGA synthesis; in fact, PgsA may form a complex with PgsE [31], although its function is unclear. A transgenic tobacco plant expressing *pgsB*, *pgsC*, and *pgsA* was capable of synthesizing γ-PGA [32], and a similar observation was made in *Escherichia coli* cells expressing these genes from *B. subtilis* [33].

*capB*, *capC*, *capA*, and *capE* in *B. anthracis* are highly similar to *pgsBCAE* in *B. subtilis*. DNA sequence analysis has revealed that three of these genes (*pgsBCA* and *capBCA*) have high sequence similarity and are located in the same γ-PGA operon (Figure 2) [31,34]. *capBCAE* encodes components of the membrane γ-PGA synthase complex. PgsB and PgsC (CapB and CapC) are responsible for γ-PGA polymerization, whereas PgsA and PgsE (CapA and CapE) mediate γ-PGA transport [30,31]. The ATPase activity of PgsB and PgsC catalyzes γ-PGA polymerization. This activity is further enhanced in the presence of PgsA [35]. PgsB has a highly hydrophobic group at one end of the protein as well as smaller membrane-binding domains. PgsC also has a highly hydrophobic group that interacts with the cell membrane. PgsA has three domains including a highly hydrophobic membrane-binding segment that contributes to γ-PGA transport [35]. However, the role of PgsE protein in γ-PGA synthesis remains unclear. PgdS (previously named YwtD) is a γ-d,l-glutamyl hydrolase that degrades γ-PGA in *B. subtilis* [36] by cleaving the γ-glutamyl bond between d- and l-glutamic acids.

**Figure 3:**
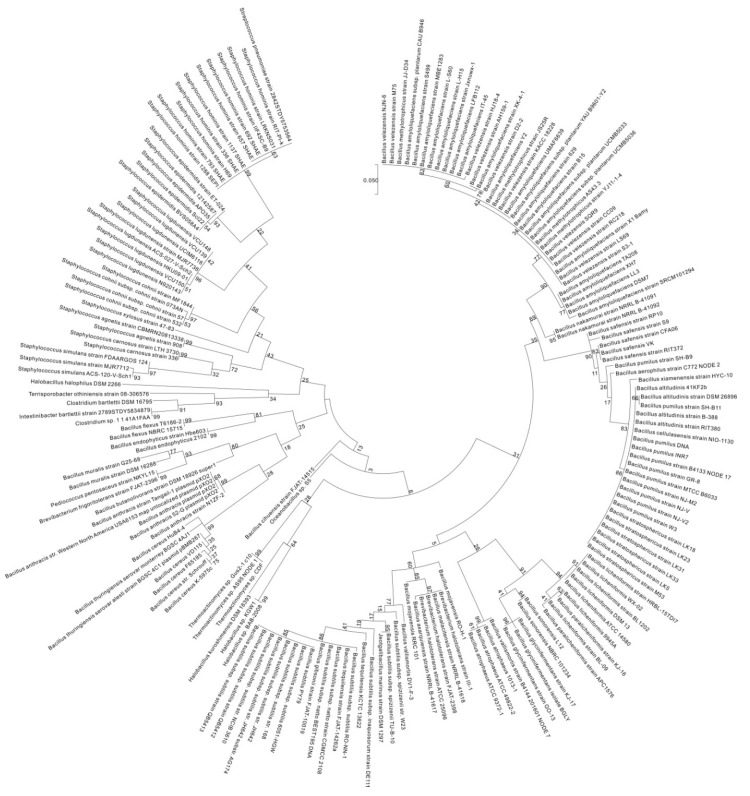
Molecular phylogenetic analysis of *pgsB* by the maximum likelihood method. The phylogenetic tree with branch lengths was measured as the number of substitutions per site. The analysis involved 192 nucleotide sequences. Partial deletion was used, and all positions with less than 95% site coverage were eliminated, i.e., less than 5% of the alignment gaps, instances of missing data, and ambiguous bases were allowed at any position. Scale bar: number of substitutions per site. Evolutionary analyses were conducted in MEGA7 [38].

To investigate the diversity of *pgsBCAE* and *pgdS* among bacteria, we used the maximum likelihood method based on the Tamira-Nei model [37] to compare *pgsBCAE* and *pgdS* of 192 representative strains from the *Firmicutes* phylum (from National Center for Biotechnology Information) with those of the γ-PGA-positive strain *B. subtilis* 3610. By incorporating the neighbor joining method and BioNJ algorithm into the construction of a pairwise distance matrix, which is calculated based on the maximum composite likelihood (MCL) function, the initial trees used for heuristically searching against multiple sequences were obtained systematically [38]. Then, the phylogenetic topology with superior log likelihood value was determined. *pgsB* was found to be broadly existed among *Bacillus* species, although it was absent in *B. megaterium*, *B. cereus*, and *B. thuringiensis* and showed low conservation in other genera. The gene was conserved in *Halotolerans* (*Brevibacteria*) strains ATCC 25096 and FJAT-2398 (Table S1 and Figure 3) as well as in several *Staphylococcus* strains (74–80% similarity) (Table S1).

**Figure 4:**
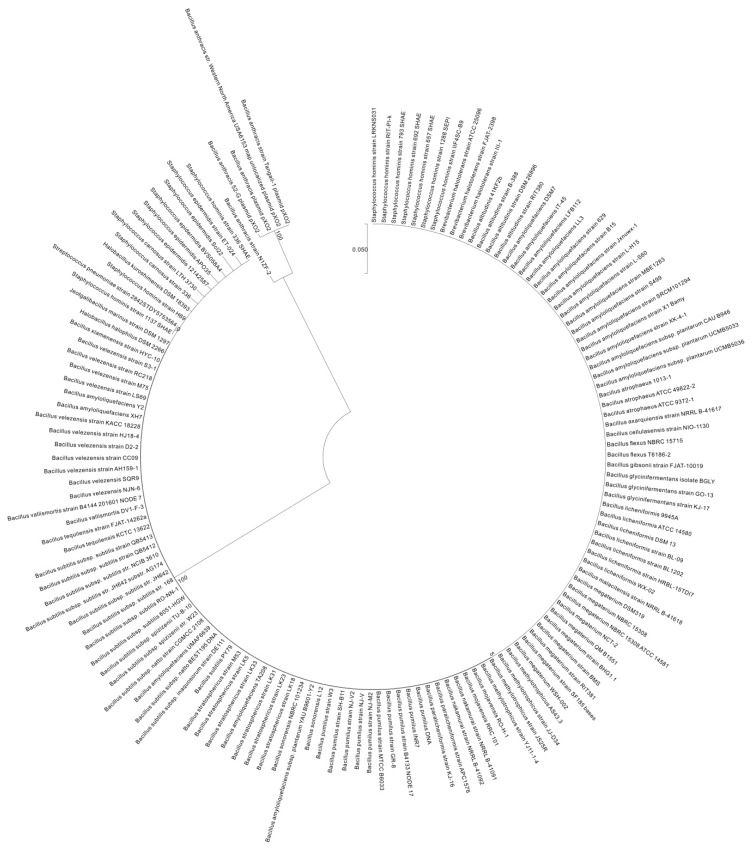
Molecular phylogenetic analysis of *pgsC* by the maximum likelihood method. The phylogenetic tree with branch lengths was measured as the number of substitutions per site. The analysis involved 192 nucleotide sequences. Partial deletion was used and all positions with less than 95% site coverage were eliminated, i.e., less than 5% of the alignment gaps, instances of missing data, and ambiguous bases were allowed at any position. Scale bar: number of substitutions per site. Evolutionary analyses were conducted in MEGA7 [38].

**Figure 5:**
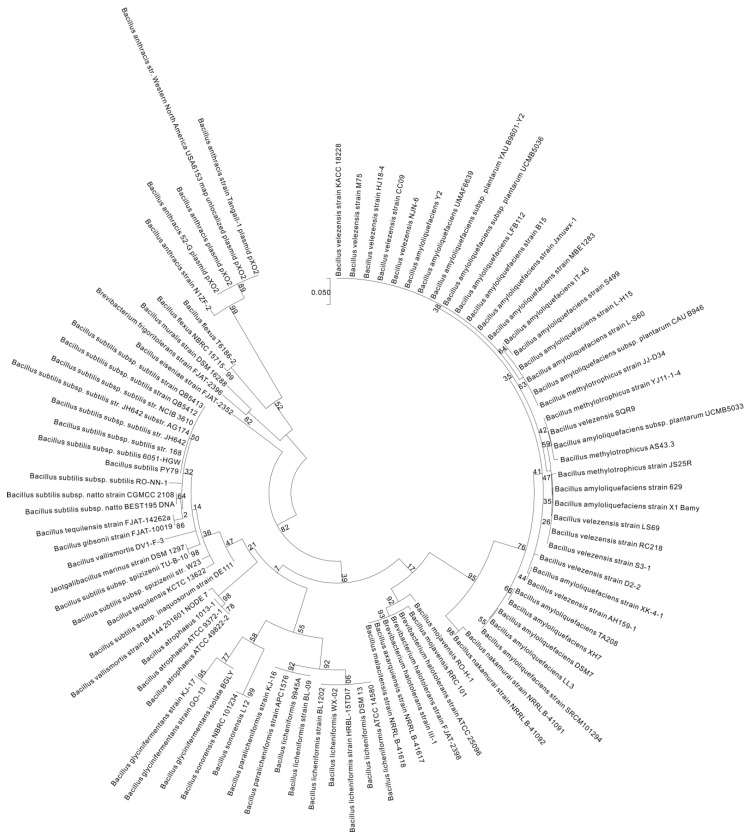
Molecular phylogenetic analysis of *pgsA* by the maximum likelihood method. The phylogenetic tree with branch lengths was measured as the number of substitutions per site. The analysis involved 192 nucleotide sequences. Partial deletion was used and all positions with less than 95% site coverage were eliminated, i.e., less than 5% of the alignment gaps, instances of missing data, and ambiguous bases were allowed at any position. Scale bar: number of substitutions per site. Evolutionary analyses were conducted in MEGA7 [38].

**Figure 6:**
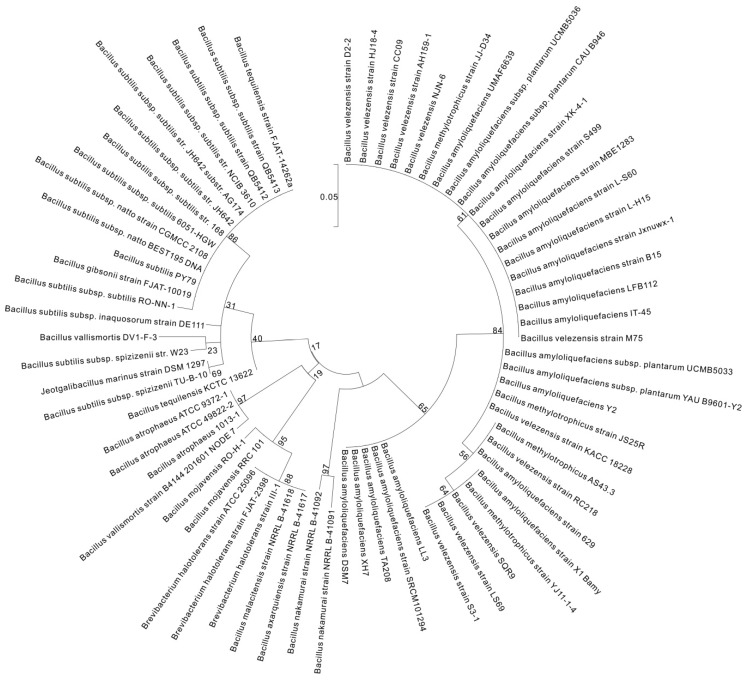
Molecular phylogenetic analysis of *pgsE* by the maximum likelihood method. The phylogenetic tree with branch lengths was measured as the number of substitutions per site. The analysis involved 192 nucleotide sequences. Partial deletion was used and all positions with less than 95% site coverage were eliminated, i.e., less than 5% of the alignment gaps, instances of missing data, and ambiguous bases were allowed at any position. Scale bar: number of substitutions per site. Evolutionary analyses were conducted in MEGA7 [38].

*pgsC* broadly existed among *Bacillus* spp., although it was absent in *B. aerophilus*, *B. altitudinis*, *B. butanolivorans*, *B. cereus*, *B. cihuensis*, *B. endophyticus*, *B. eiseniae*, *B. safensis*, and *B. thuringiensis* and most other *non-Bacillus* species (Table S1 and Figure 4). *pgsA* existed in *Bacillus*, but was absent in *B. aerophilus*, *B. altitudinis*, *B. butanolivorans*, *B. cellulasensis*, *B. cereus*, *B. cihuensis*, *B. endophyticus*, *B. eiseniae*, *B. megaterium*, *B. muralis*, *B. safensis*, *B. stratosphericus*, *B. thuringiensis*, and *B. xiamenensis* and most other genera (Table S1 and Figure 5). *pgsE* did not broadly exist across *Bacillus* species, although it was present in *B. amyloliquefaciens*, *B. atrophaeus*, *B. axarquiensis*, *B. gibsonii*, *B. malacitensis*, *B. methylotrophicus*, *B. nakamurai*, *B. subtilis* subsp. *natto*, *B. subtilis* subsp. *subtilis*, *B. subtilis* subsp. *spizizenii*, and *B. velezensis* as well as in *Halotolerans strains* (Table S1 and Figure 6).

**Figure 7:**
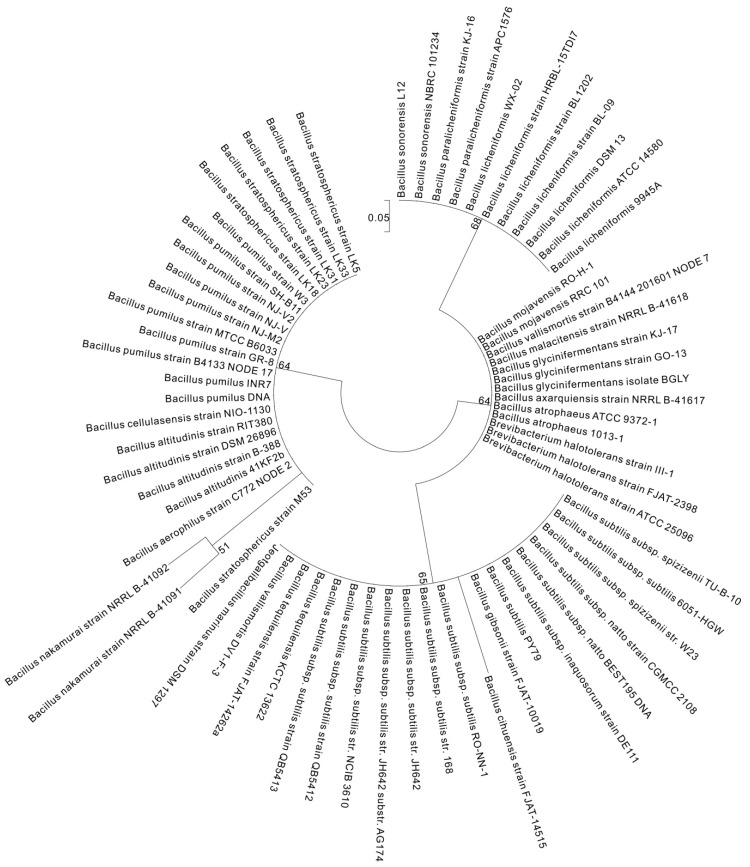
Molecular phylogenetic analysis of *pgdS* by the maximum likelihood method. The phylogenetic tree with branch lengths was measured as the number of substitutions per site. The analysis involved 192 nucleotide sequences. Partial deletion was used and all positions with less than 95% site coverage were eliminated, i.e., less than 5% of the alignment gaps, instances of missing data, and ambiguous bases were allowed at any position. Scale bar: number of substitutions per site. Evolutionary analyses were conducted in MEGA7 [38].

**Figure 8:**
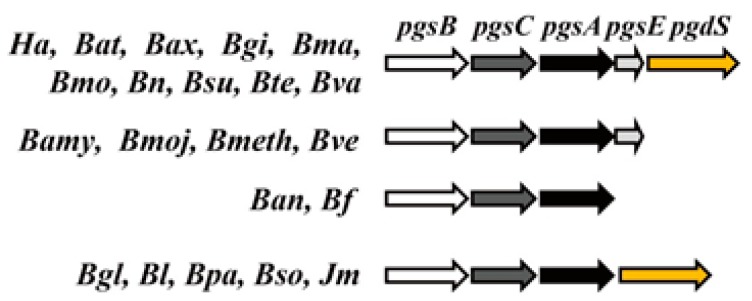
*pgsBCAE* and *pgdS* gene clusters. *Bat*, *Bacillus atrophaeus*; *Bax*, *Bacillus axarquiensis*; *Bgi*, *Bacillus gibsonii*; *Bma*, *Bacillus malacitensis*; *Bmo*, *Bacillus mojavensis*; *Bn*, *Bacillus nakamurai*; *Bsu*, *Bacillus subtilis*; *Bte*, *Bacillus thuringiensis*; *Bva*, *Bacillus vallismortis*; *Bamy*, *Bacillus amyloliquefaciens*; *Bmoj*, *Bacillus mojavensis*; *Bmeth*, *Bacillus methylotrophicus*; *Bve*, *Bacillus velezensis*; *Ban*, *Bacillus anthracis*; *Bf*, *Bacillus flexus*; *Bgl*, *Bacillus glycinfermentants*; *Bl*, *Bacillus licheniformis*; *Bpa*, *Bacillus paralicheniformis*; *Bso*, *Bacillus sonorensis*; *Ha*, *Halotolerans*; *Jm*, *Jeotgalibacillus marinus*.

The *pgdS* gene did not broadly exist in the Bacillus strains but was found in *B. amyloliquefaciens*, *B. atrophaeus*, *B. axarquiensis*, *B. gibsonii*, *B. licheniformis*, *B. malacitensis*, *B. mojavensis*, *B. nakamurai*, *B. paralicheniformis*, *B. subtilis* subsp. *natto*, *B. subtilis* subsp. *subtilis*, *B. subtilis* subsp. spizizenii, and *B. vallismortis* as well as in *Jeotgalibacillus marinus*, and *Halotolerans strains* (Table S1 and Figure 7). The fact that *B. anthracis* and other species do not harbor *pgsE* (Figure 8) may explain the fact that it does not secrete γ-PGA, which is instead anchored to the cell membrane. On the contrary, in species lacking *pgdS*, γ-PGA degradation may not proceed efficiently. The results of the phylogenetic diversity revealed that the conservation of genes decreased in the following order: *pgsC* > *pgdS* > *pgsE* > *pgsA* > *pgsB* (Figure 3, Figure 4, Figure 5, Figure 6 and Figure 7). *pgsC* is important for γ-PGA synthesis, and thus, it is difficult to modify or replace it, but *pgsB*, *pgsA*, and *pgsE* can vary or be replaced without affecting γ-PGA synthesis.

## 4. Regulation of the *pgsBCA* Operon

**Figure 9:**
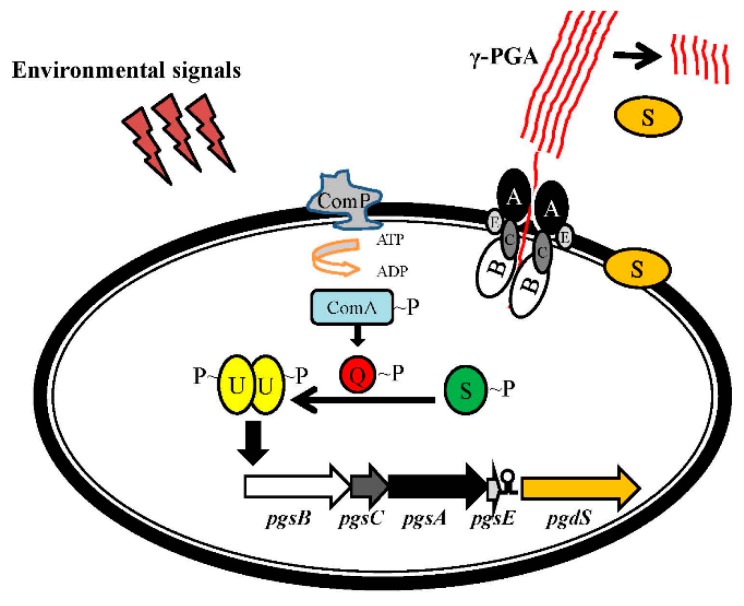
Regulation of the *pgsBCA* gene cluster: A, *pgsA*; B, *pgsB*; C, *pgsC*; E, *pgsE*; U, *degU*; Q, *degQ*; S, *degS*.

*pgsBCA* cluster is regulated by the two-component DegS–DegU system and a ComP–ComA quorum sensing system [39,40] (Figure 9). These two systems activate the transcription of *pgsBCA* in response to changes in environmental osmolality and phase. At high cell density, phosphorylated ComP protein phosphorylates ComA, ComA–P activates DegQ, and DegQ–P promotes the phosphorylation of DegS and DegU. DegU–P level regulates *pgsBCA* expression.

It has been shown that cooperation between SwrA, a protein needed for *Bacillus* spp. swarming, and DegU–P is required to fully activate the *pgsBCA* operon [41]. This genetic circuitry responds to signals of quorum sensing, osmolality, high cell density, and phase variation; it indicated that these environmental signals control γ-PGA synthesis [40]. Deletion of *degQ* markedly decreased γ-PGA synthesis in B. subtilis strains [40,42]. It was also reported that deletion of the motility genes *motA* and *motB* increased γ-PGA production [43]. These findings suggest that inhibiting flagellar rotation may induce DegU–P expression and increase γ-PGA production (Figure 9).

## 5. Recombinant Strains Used for γ-PGA Production

Genetically engineered recombinant strains, including Δ*pgdS*Δ*cwlO*, Δ*cwlO*Δ*epsA-O*, Δ*pgdS*Δ*ggt*, and Δ*rocR*Δ*rocG*Δ*gudB*Δ*odhA* deletion mutants and strains overexpressing *pgdS*, *pgsBCA*, *pgsE*, and *glr*; short RNA corresponding to *rocG*; or antagonists of *rocG* and *glnA*, have been used to produce large amounts of γ-PGA (Table 2). The γ-PGA yields of these strains are 2 to 10 times higher than those of the wild-type strains.

*pgdS*, *cwlO*, and *ggt* are involved in γ-PGA degradation [15,36,54]. *odhA* and *odhB* encode components of the 2-oxoglutarate dehydrogenase complex that catalyzes the oxidative decarboxylation of 2-oxoglutarate to succinyl coenzyme (CoA) in *B. subtilis* [55], which increases the flux from 2-oxoglutarate to glutamate. *rocG* encodes glutamate dehydrogenase in *B. subtilis*, which is associated with glutamate degradation [56]. *rocR* is also involved in glutamate metabolism in *Bacillus* species. Deletion of *rocR*, *rocG*, *gudB*, and *odhA* increase the intracellular glutamate concentration, which facilitates γ-PGA production [46]. The *epsA-O* gene cluster regulates the production of exopolysacharrides (EPS) [57,58], which are the main byproducts of some γ-PGA-producing strains. 

Thus, deletion of *epsA-O* can inhibit EPS production, whereas metabolic flux can be used to enhance γ-PGA productivity. *glr* encodes glutamate racemase, which is involved in the conversion of l-glutamic acid to its d isomer [59], thereby increasing γ-PGA yield [50]. A similar role is attributed to glutamine synthetase encoded by *glnA* [53].

The *B. subtilis* PB5249 Δ*pgdS*Δ*ggt* mutant produced 40 g/L γ-PGA, which was twice the yield produced by the wild-type strain [47]. *B. licheniformis* WX-02 transfected with the pHY300PLK-PgdS-*pgdS* plasmid of two constructs produced 20.16 g/L γ-PGA [51]. pWPSE-*xylA*-*pgsE* overexpression in *B. subtilis* subsp. *chungkookjang* yielded the lowest amount of γ-PGA among all transgenic strains (0.6 g/L) [49]. Mutation of the *B. subtilis pgsE* is a strategy for increasing γ-PGA yield without altering the structural features of the protein [49]; additionally, overexpression of *pgsBCA* in *B. subtilis* WB600 increased γ-PGA production by approximately 10-fold relative to the wild-type strain, although the yield was only 1.74 g/L [48]. Thus, although γ-PGA production is increased in recombinant strains, the culture medium must be supplemented with antibiotics to maintain the enhanced or disrupted genes, which increases the cost of fermentation.

## 6. Fermentation Conditions

Most strains used for γ-PGA production are *Bacillus* species (Table 3), with some strains producing higher amounts under fermentation conditions [60,61]. In most cases, flasks or fermenters are used as a bioreactor, with the highest yields obtained with the former. Several studies have directly compared γ-PGA yield under the two conditions.

Carbon sources, such as glucose, citric acid, and other sugars, are used for γ-PGA production. Glutamate is used as a nitrogen source or as a reaction substrate, whereas metals, such as zinc, are used to induce γ-PGA production. Glucose and glutamate are the most widely used carbon and nitrogen sources, respectively, for fermentation with *B. subtilis* NX-2, ZJU-7, CGMCC833, and P-104 strains (Table 3). *B. subtilis* ZJU-7 showed the highest γ-PGA yield, which reached 101.1 g/L using glucose and glutamate as substrates; *B. subtilis* NX-2 showed the lowest yield of 31.7 g/L [60,62].

Several strains generated for γ-PGA production use citric acid with glutamate as the main substrates. *B. licheniformis* NCIM2324 had the highest γ-PGA yield of 98.64 g/L [63], whereas the other *B. licheniformis* ATCC9945 strain had the lowest yield of 12.64 g/L [64]. The use of other sugars, such as sucrose, xylose, cane molasses, or mannitol, supplemented with glutamate as the fermentation substrate further improved γ-PGA production, with *B. subtilis* NX-2 (73.0 g/L) and *B. subtilis* GXA-28 (8.72 g/L) yielding the highest and lowest amounts, respectively [65,66]. With glucose and NH_4_Cl as substrates, *B. licheniformis* A13 produced the highest amount of γ-PGA at 28.2 g/L, whereas *B. licheniformis* TISTR 1010 produced the lowest at 27.5 g/L [67,68].

Finally, *B. subtilis* NX-2 yielded the highest amount of γ-PGA at 107.7 g/L in flask cultures, which was comparable to the yield using glutamate with different sugars as substrates under fermentation conditions. The lowest amount produced with sucrose as substrate was by *B. subtilis* GXA-28 (8.72 g/L under flask culture conditions). 

Of all the strains shown in Table 3, *B. subtilis* NX-2 produced the highest amount of γ-PGA, demonstrating the potential of this strain for large-scale γ-PGA production. However, this requires a medium that includes oyster, shiitake, needle, or eryngii mushroom or *Agaricus bisporus* residue as well as different carbon sources. *B. licheniformis* NCIM 2324 has also been used as a fermentation starter, with a maximum γ-PGA yield of 98.64 g/L in the presence of l-glutamic acid and citric acid. Glucose enters glycolysis followed by the TCA cycle; however, citric acid is a TCA cycle intermediate that is converted to l-glutamic acid, a substrate for γ-PGA synthesis. Therefore, γ-PGA production can possibly be enhanced by the inclusion of both the nutrients in the medium.

## 7. Applications of γ-PGA

γ-PGA has been used as an antifreeze, thickener, food and feed additive, and humectant, and its ester derivatives can be used in biologically degradable materials, such as heat-resistant plastic film and fibrous substitutes [6,20,87,88,89]. Recent studies have highlighted other potential applications of γ-PGA. As a biopolymer that is water-soluble, biodegradable, resistant to moisture, and non-toxic, and that has strong adsorption properties, high viscosity, and metal-chelating capacity, γ-PGA is an environmentally safe polymer material [5,9,10,89,90,91,92,93,94] for use in cosmetics, food, pharmaceutical, and other industries [3,4]. γ-PGA derivatives have high water-absorption capacity as a flocculating agent and can be used as a nutritional additive to promote calcium absorption for prevention of osteoporosis, wetting agent, and biological adhesive [4,5,88,95]. In the food industry, γ-PGA can be used as an additive to increase the viscosity of liquid foods and as a stabilizer to improve the texture and taste of baked, fried, or frozen foods and beverages [6,7,8]. In cosmetics, γ-PGA can act as a humectant that improves skin maintenance and reduces wrinkles [89,96]. In sewage treatment, γ-PGA can remove heavy metal ions and radioactive elements owing to its high metal-chelating properties [9,10]. Using a low-pressure ultrafiltration technique, it has been found that γ-PGA binds to and efficiently removes >99.8% of lead ions [97]. With a molecular weight of ~5.8–6.2 × 10^6^ Da, γ-PGA is much better than many conventional flocculants for industrial wastewater treatment because it is ecofriendly [94]. Interestingly, γ-PGA with a molecular weight of 9.9 × 10^5^ Da efficiently removed 98% of basic dyes from aqueous solution at pH 1.0, and was reusable [10,90]. The biodegradability of γ-PGA makes it suitable for use as a drug or gene carrier in biomedical applications [3,4,5,32]. In addition, γ-PGA has high water absorption capacity, and therefore, can improve soil and seed coatings and balance soil pH, thereby increasing agricultural productivity. Finally, γ-PGA can strengthen the effects of biological pesticides in the control of plant pests and diseases [98].

Although γ-PGA has many potential applications, there are various limitations that must be overcome for its widespread use. First, the cost of production of γ-PGA is much higher than that of conventional thermoplastic materials; given the difficulty of chemical γ-PGA synthesis, generating genetically engineered strains with higher γ-PGA productivity is important.

## 8. Conclusions

In addition to its several advantageous properties, γ-PGA is mainly produced by non-harmful bacteria, and therefore, can be safely used in industrial applications. γ-PGA fermentation or production can be achieved using different strains, substrates, and bioreactors through various processes. In addition to other biological parameters, controlling culture pH is important for maximizing yield [99]. Moreover, comparing high-yield γ-PGA strains reported in various studies under the same growth conditions can help identify the strains that are the most efficient fermentation starters. Our phylogenetic analysis provides a broad view of *Bacillus* species that harbor full *pgsBCAE* and *pgdS*. This information can aid in the selection of candidate strains for genetic manipulation to maximize γ-PGA production. The observed variation in *pgsBCAE* and *pgdS* among γ-PGA-producing *Bacillus* strains may explain why some strains do not degrade γ-PGA, as they lack *pgdS*, and why some strains export γ-PGA, whereas those that lack *pgsE* do not, as PgsE is thought to interact with PgsA in this process. This will help in selection of strains for high production of γ-PGA and in genetic engineering of selected γ-PGA production strains. It will also help us fully understand how to manipulate PGA synthesis at the molecular level.

The high cost of γ-PGA production can potentially be reduced through genetic manipulation of γ-PGA related and regulatory genes. This has been attempted for several γ-PGA-producing strains, leading to increased resistance of these bacteria to environmental stresses and improved γ-PGA yields. Few strains are considered useful for industrial-scale γ-PGA production. Future studies can explore whether other bacterial species or different microorganisms can be used for this purpose, given the importance and multiple potential uses of γ-PGA.

## Figures and Tables

**Table 1:** d/l forms of γ-PGA-producing strains.

γ-PGA	Composition (%)	Strains	Reference
d-Glutamate	100	*Bacillus anthracis*	[11]
l-Glutamate	100	*Natrialba aegyptiaca*	[24]
d-/l-Glutamate	60/40	*Bacillus subtilis*	[28,29]
	10–100/10–90	*Bacillus licheniformis*	[25,26]
	30/70	*Bacillus megaterium*	[27]
	40/60	*Staphylococcus epidermidis*	[23]

**Table 2:** Recombinant *Bacillus* strains generated for increased γ-PGA production.

Strains	Genotype	Fermentation Medium	Wild-Type Yield (g/L)	Yield (g/L)	Increasing Yield (%)	Reference
*B. amyloliquefaciens* LL3	Δ*pgdS*Δ*cwlO*	Sucrose, (NH_4_)_2_SO_4_, MgSO_4_, KH_2_PO_4_, K_2_HPO_4_	3.69	7.12	92.95	[44]
*B. amyloliquefaciens*	Δ*cwlO*Δ*epsA-Ovgb*	Sucrose, (NH_4_)_2_SO_4_, MgSO_4_, KH_2_PO_4_, K_2_HPO_4_, trace elements (FeSO_4_·4H_2_O, CaCl_2_·2H_2_O, MnSO_4_·4H_2_O, ZnCl_2_)	3.14	5.12	63.06	[45]
*B. amyloliquefaciens* LL3	Δ*rocR*Δ*rocG*Δ*gudB*Δ*odhA*	Sucrose, (NH_4_)_2_SO_4_, MgSO_4_, KH_2_PO_4_, K_2_HPO_4_	4.03	5.68	40.94	[46]
*B. subtilis* PB5249	Δ*pgdS*Δ*ggt*	l-glutamate, citric acid, glucose, NH_4_Cl, K_2_HPO_4_, MgSO_4_·7H_2_O, FeCl_3_·6H_2_O, CaCl_2_·2H_2_O, MnSO_4_·H_2_O	20	40	100	[47]
*B. subtilis* WB600	pWB980-*pgsBCA*	Glucose, sodium glutamate, (NH_4_)_2_SO_4_, K_2_HPO_4_, MgSO_4_	0.134	1.74	1198.51	[48]
*B. subtilis* subsp. *chungkookjang*	pWPSE-P*_xylA_*-*pgsE*	l-glutamate, sodium citrate, casamino acid, yeast extract, (NH_4_)_2_SO_4_, MgCl_2_, Na_2_HPO_4_, KH_2_PO_4_, NaCl	0.20	0.64	220	[49]
*B. licheniformis* WX-02	pHY300PLK-P_43_-*glr*	Sucrose, (NH_4_)_2_SO_4_, MgSO_4_, KH_2_PO_4_, K_2_HPO_4_	11.73	14.38	22.59	[50]
*B. licheniformis* WX-02	pHY300PLK-P*_pgdS_*-*pgdS*	Glucose, sodium glutamate, sodium citrate, NH_4_Cl, MgSO_4_, K_2_HPO_4_, CaCl_2_, ZnSO_4_, MnSO_4_	13.11	20.16	53.78	[51]
*B. subtilis* ISW1214	pWH1520-P*_xylA_*-*pgsBCA*	Sucrose, NaCl, MgSO_4_, KH_2_PO_4_, NaHPO_4_, xylose	8.2	9.0	9.76	[52]
*B. amyloliquefaciens*	sRNA of *rocG* (repressed *rocG* and g*lnA* genes)	Sucrose, (NH_4_)_2_SO_4_, MgSO_4_, KH_2_PO_4_, K_2_HPO_4_	14.96	20.3	35.69	[53]

**Table 3:** γ-GPA fermentation strains, fermentation recipe, process control, and yield.

Main Substrate	Strain	Recipe	Fermentation Conditions	Flask/Fermenter	Yield (g/L)	Reference
Glucose + Glutamate	*B. subtilis* NX-2	Glucose, l-glutamate, MgSO_4_, K_2_HPO_4_·3H_2_O, NH_4_SO_4_, MnSO_2_	7.5-L bioreactor, 400 rpm, 1.2 vvm, pH 7.0, 32 °C	Fermenter	71.21	[69]
	*B. subtilis* NX-2	Glucose, l-glutamate, Glycerol, K_2_HPO_4_·3H_2_O, MgSO_4_, (NH_4_)_2_SO_4_, MnSO_4_	500-mL flask, 220 rpm, pH 7.5, 32.5 °C	Flask	31.7	[62]
	*B. subtilis* NX-2	Glucose, l-glutamate, K_2_HPO_4_·3H_2_O, MgSO_4_, NH_4_Cl	110-L bioreactor, 220 rpm, pH 7.5, 32.5 °C	Fermenter	35.0	[70]
	*B. subtilis* NX-2	Glucose, l-glutamate, (NH_4_)_2_SO_4_, K_2_HPO_4_·3H_2_O, MgSO_4_, MnSO_4_	7.5-L BioFlo 110 bioreactor, 200–800 rpm, 1.5 vvm, pH 7.0, 32 °C	Fermenter	40.5	[71]
	*B. subtilis* ZJU-7	Glucose, l-glutamate, yeast extract, NaCl, CaCl_2_, MgSO_4_, MnSO_4_	10-L bioreactor, 300–800 rpm, 1.5 vvm, pH 6.5, 37 °C	Fermenter	101.1	[60]
	*B. subtilis* ZJU-7	Glucose, l-glutamate, tryptone, NaCl, MgSO4, CaCl2	500-mL flask, 200 rpm, pH 7.0, 37 °C	Flask	58.2	[65]
	*B. subtilis* CGMCC 0833	Glucose, l-glutamate, (NH_4_)_2_SO_4_, K_2_HPO_4_·3H_2_O, MgSO_4_, MnSO_4_	7.5-L reactor, 400 rpm, pH 7.5, 32.5 °C	Fermenter	34.4	[72]
	*B. subtilis* ZJU-7	Glucose, l-glutamate, (NH_4_)_2_SO_4_, K_2_HPO_4_·3H_2_O, MnSO_4_, MgSO_4_	100-L fermenter, 200–450 rpm, 0.5–1 vvm, pH 6.5, 30 °C	Fermenter	54.0	[73]
	*B. licheniformis* P-104	Glucose, sodium glutamate, sodium citrate, (NH_4_)_2_SO_4_, MnSO_4_, MgSO_4_, K_2_HPO_4_, NaNO_3_	7-L bioreactor, 500 rpm, 1.5 vvm, pH 7.2	Fermenter	41.6	[14]
	*B. subtilis* ZJU-7	Glucose, l-glutamate, tryptone, NaCl	7.0, 37 °C, fed-batch 100 mL flask, 200 rpm, pH 7.0, 37 °C	Flask	54.4	[74]
Citrate + Glutamate	*B. licheniformis* ATCC9945	Citric acid, l-glutamate, glycerol, NH_4_Cl, K_2_HPO_4_, MgSO_4_·7H_2_O, FeCl_3_·6H_2_O, CaCl_2_·2H_2_O, MnSO_4_·H_2_O	225 mL flask, 250 rpm, pH 6.5, 30 °C	Flask	12.64	[64]
	*B. subtilis* MJ80	Citric acids, l-glutamate, starch, urea, glycerol	300 L fermenter, 150 rpm, 1 vvm, initial pH 7.0, 37 °C	Fermenter	68.7	[75]
	*B. licheniformis* NCIM 2324	Citric acid, l-glutamate, glycerol, (NH_4_)_2_SO_4_, K_2_HPO_4_, MgSO_4_·7H_2_O, MnSO_4_·7H_2_O, CaCl_2_·2H_2_O, MnSO_4_·7H_2_O	250-mL flask, 200 rpm, initial pH 6.5, 37 °C	Flask	35.75	[76]
	*B. licheniformis* NCIM 2324	Citric acids, l-glutamate, MgSO_4_·7H_2_O, MnSO_4_·2H_2_O α-ketoglutaric acid	250-mL Erlenmeyer flask, 200 rpm, pH 7.0, 37 °C	Flask	98.64	[63]
	*B. subtilis* BL53	Citric acid, l-glutamate, glycerol, NH_4_Cl, MgSO_4_·7H_2_O, FeCl_3_·6H_2_O, K_2_HPO_4_, CaCl_2_·2H_2_O, MnSO_4_·H_2_O	250-mL flask, 180 rpm, pH 6.5, 37 °C	Flask	17.0	[77]
	*B. licheniformis* NCIM 2324	Citric acids, l-glutamate glycerol, ammonium sulphate	250-mL flasks, 200 rpm, pH 5–8, 37 °C	Flask	26.12	[17]
	*B. licheniformis* NCIM 2324	Citric acid, l-glutamate, glycerol, ammonium sulfate, α-ketoglutaric acid, K_2_HPO_4_, MgSO_4_·7H_2_O, CaCl_2_·2H_2_O, MnSO_4_·7H_2_O	Biostat B5 fermenter (2.5 L), 250–1000 rpm, 1.0–3.0 vvm, pH 6.5, 37 °C	Fermenter	46.34	[78]
	*B. licheniformis* ATCC 9945	Citric acid, l-glutamate, glycerol, NH_4_Cl, K_2_HPO_4_, MgSO_4_·7H_2_O, FeCl_3_·6H_2_O, CaCl_2_·2H_2_O, MnSO_4_·H_2_O	500-mL Erlenmeyer flask, pH 7.4, 37 °C	Flask	35.78	[79]
	*B.* sp. SW1-2	Citric acids, l-glutamate, glycerol, K_2_HPO_4_, MgSO_4_·7H2O, FeSO_4_·4H_2_O, CaCl_2_·2H_2_O, MnSO_4_·4H_2_O, ZnCl_2_, NH_4_)_2_SO_4_, casein hydrolysate.	250-mL Erlenmeyer flask, 150 rpm, pH 7.0, 37 °C	Flask	36.5	[80]
Other sugars + Glutamate	*B. subtilis* NX-2	l-glutamate, (NH_4_)_2_SO_4_, K_2_HPO_4_, MgSO_4_, MnSO_4_, carbon source (glucose, xylose)	7.5-L bioreactor, 400 rpm, 1.2 vvm, initial pH 7.0, 32 °C	Fermenter	73.0	[61]
	*B. subtilis* HB-1	l-glutamate, xylose, yeast extract, NaCl, MgSO_4_, CaCl_2_,	10-L bioreactor, 500 rpm, initial pH 6.5, 37 °C, fed-batch	Fermenter	28.15	[66]
	*B. subtilis* NX-2	Cane molasses, glutamate, (NH_4_)_2_SO_4_, wet cells, crude protein, reducing sugar, potassium, calcium, magnesium, manganese, iron, phosphonium.	7.5-L bioreactor, 400 rpm, 1.2 vvm, pH 7.0, 32 °C	Fermenter	52.1	[81]
	*B. subtilis* D7	l-glutamate acid, mannitol, yeast extract, K_2_HPO_4_, NaH_2_PO_4_, MgSO_4_·7H_2_O, CuSO_4_·5H_2_O, pyruvic acid	300-mL Erlenmeyer flask, 250 rpm, pH 8.0, 35 °C	Flask	24.93	[82]
	*B. subtilis* GXA-28	soybean residue, sucrose, glutamate	250-mL flask, 200 rpm, pH 2.0–10.0, 45 °C	Flask	8.72	[83]
Glucose + NH_4_Cl	*B. subtilis* C10	Glucose, NH_4_Cl, MgSO_4_·7H_2_O, K_2_HPO_4_, FeCl_3_·6H_2_O, MgSO_4_·H_2_O, CaCl_2_, CaCO_3_	10-L fermenter, 200–500 rpm, 1.5 vvm, pH 7.5, 32 °C	Fermenter	27.70	[84]
	*B. licheniformis* A13	Glucose, NH_4_Cl, NaCl, MgSO_4_·7H_2_O, CaCl_2_·2H_2_O, K_2_HPO_4_, FeSO_4_·4H_2_O, NaMoO_4_, CuSO_4_, MnSO_4_, ZnSO_4_, CoCl_2_, H_3_BO_4_	250-mL Erlenmeyer flask, 200 rpm, pH 6.5, 37 °C	Flask	28.2	[67]
	*B. licheniformis* TISTR 1010	Glucose, citric acid, NH_4_Cl, K_2_HPO_4_, MgSO_4_·7H_2_O, FeCl_3_·6H_2_O, CaCl_2_·2H_2_O, MnSO_4_·H_2_O, NaCl, Tween-80	7-L fermenter, 300 rpm, 1 vvm, pH 7.4, 37 °C	Fermenter	27.50	[68]
Others	*B. methylotrophicus*	Glucose, yeast extract, MgSO_4_·7H_2_O, K_2_HPO_4_ MnSO_4_	250-mL flasks, 200 rpm, pH 7.2, 37 °C	Flask	35.34	[85]
	*B. subtilis* NX-2	Glucose, cane molasses, xylose, starch, industrial waste glycerol, citric acid, DMR, MGPR (oyster, shiitake, needle, eryngii mushroom, and Agaricus bisporus residues	500-mL shake flask, 150 rpm, pH 7.0, 35 °C	Flask	107.7	[86]

## Supplementary Materials

The following are available online at www.mdpi.com/1422-0067/18/12/2644/s1.

## Abbreviations

γ-PGA	Poly-γ-glutamic acid
TCA	Tricarboxylic acid
ATP	Adenosine triphosphate
MCL	Maximum composite likelihood
CoA	Coenzyme
EPS	Exopolysaccharides
